# Acute Scrotum: A Rare Manifestation of Henoch-Schönlein Purpura

**DOI:** 10.7759/cureus.96655

**Published:** 2025-11-12

**Authors:** Taofiq Olayinka Mohammed, Abdirashid Hassan Kassim, Anthony Blacker

**Affiliations:** 1 Urology, University Hospitals Coventry and Warwickshire National Health Services (NHS) Trust, Coventry, GBR

**Keywords:** acute scrotum, henoch-schonlein purpura, iga vasculitis (igav), testicular pain, testicular torsion mimic

## Abstract

Henoch-Schönlein purpura (HSP) is a systemic vasculitis commonly seen in children. The disease is characterised by a tetrad of clinical manifestations, which includes palpable purpura, arthritis/arthralgia, abdominal pain, and kidney involvement. Henoch-Schönlein purpura scrotal involvement is rare. They can mimic an acute scrotum and require prompt diagnosis and treatment to avert significant complications. We present the case of a six-year-old male child with HSP presenting with an acute scrotum. This case report aims to highlight the diagnostic challenges and treatment approaches for HSP-associated scrotal involvement.

## Introduction

Henoch-Schönlein purpura (HSP) is the most common form of systemic vasculitis affecting children, predominantly between the ages of 3 and 15years [1,2]. Its incidence varied widely, ranging from 3 to 7 per 100,000 population [1,3]. It is more common in male children and rare in adults. The underlying cause of IgAV is unknown. A combination of factors, including immunologic, genetic, and environmental factors, all play a role [1,4]. It is an immune-mediated vasculitis associated with IgA deposition, accompanied by complement deposition and neutrophil recruitment, often triggered by various infectious or chemical agents [4].

The disease is characterised by a tetrad of clinical manifestations, which includes palpable purpura, arthritis/arthralgia, abdominal pain, and kidney disease [1,4,5]. It is typically self-limiting, but serious complications such as renal failure can occur.

Urologic manifestation of HSP is rare. However, scrotal, ureteral, bladder, prostate, testicle, and penile involvement have been reported [6]. Testicular manifestation after HSP is very rare, and diagnosis can easily be missed.

We present the case of a six-year-old male child who developed an acute scrotum after HSP. This case report aims to raise awareness by highlighting the diagnostic challenges and treatment approaches for HSP.

## Case presentation

A six-year-old boy presented to the emergency department with a one-day history of right testicular pain, swelling, and erythema. The pain was sudden in onset, severe and persistent, and had progressively worsened. There was no history of trauma to the area. There was no haematuria and no voiding difficulty. The patient had no significant past medical history, and his immunisations were up to date. There was no family history of vasculitis or autoimmune disease. He had recently been admitted, managed, and discharged a few days earlier by the paediatric team as a case of Henoch-Schönlein purpura. He had presented with fever, intermittent abdominal pain, particularly around the umbilicus, and a non-blanching purpuric rash on the lower extremities. His clinical examination then showed stable vital signs, a soft, non-tender abdomen, and palpable purpura in the lower extremity. The urinalysis revealed trace protein only. There was no microscopic haematuria, nitrites, or leucocytes. The complete blood count, renal function, and clotting profile were all normal. He was admitted for observation. He was given analgesia and discharged a few days later following resolution of symptoms with a follow-up appointment with the paediatric nephrologist. He developed acute scrotal pain five days after discharge.

On examination, he was afebrile with stable vital signs but appeared in discomfort due to scrotal pain. The abdomen was soft and non-tender with no palpable intra-abdominal organ enlargement. The right testicle was tender, swollen, and erythematous. It was clinically difficult to exclude testicular torsion. The left testis was palpable within the left hemiscrotum and was of normal size and consistency and was non-tender.

**Figure 1:**
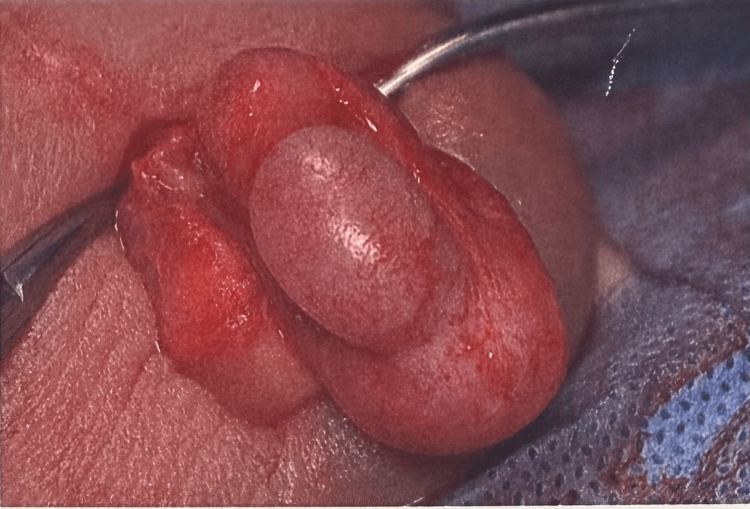
Anterior view of the right testis showing a mildly swollen right testis and a thickened oedematous right epididymis

**Figure 2:**
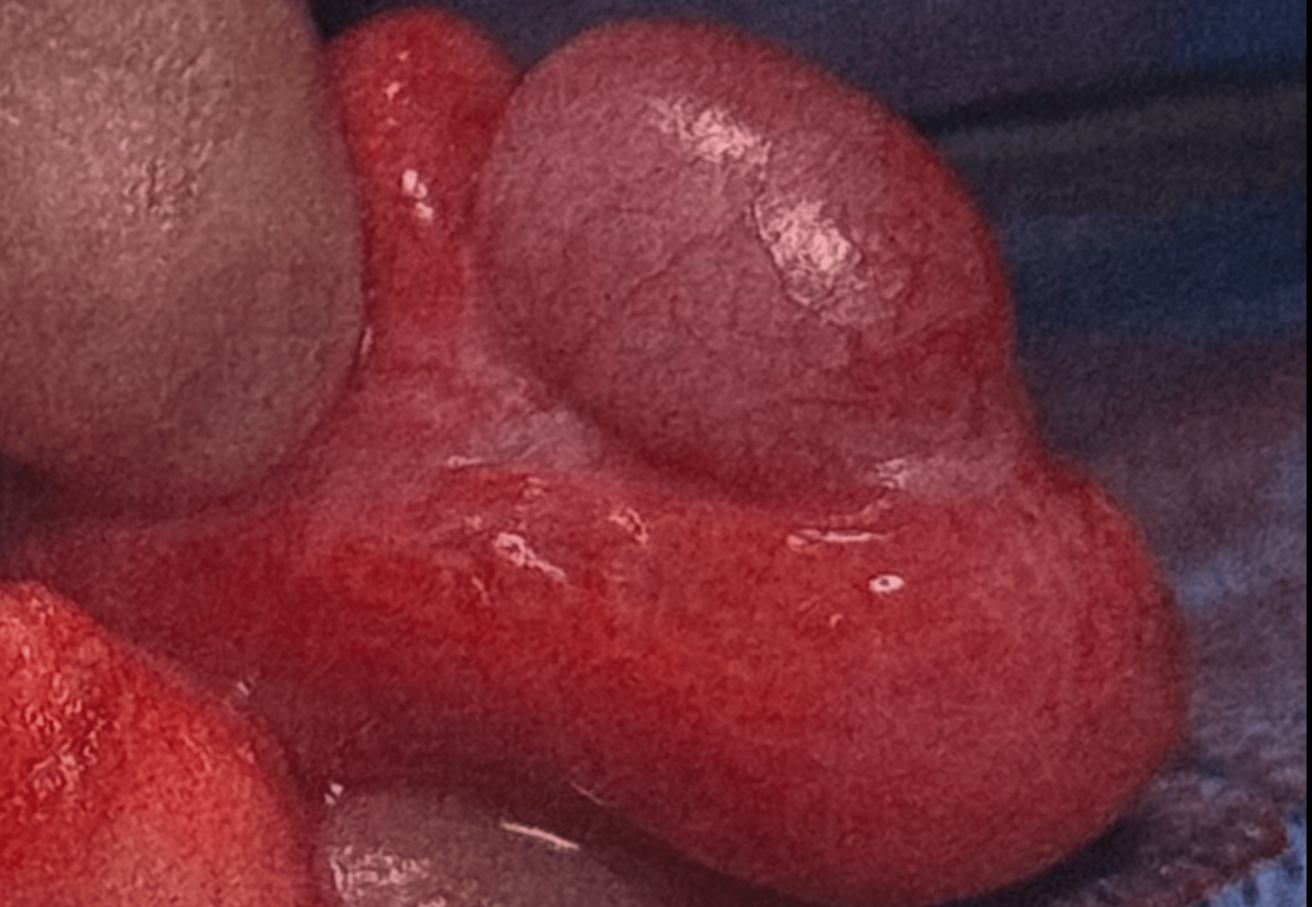
Lateral view of the right testis showing an engorged right testis and a thickened oedematous right epididymis

The purpuric rash was palpable and primarily distributed over the buttocks and legs. Blood and urinalysis were normal. Testicular ultrasound was not immediately available, and a decision was made to perform an urgent scrotal exploration to exclude testicular torsion. Intraoperative findings revealed a swollen, thickened, and oedematous epididymis and testis, a mild reactive hydrocele, vascular congestion/hyperaemia, and a slightly adherent tunica vaginalis. There was no evidence of testicular torsion. The testis was returned to the scrotum without fixation, and the scrotal wound was closed. The contralateral testis was not explored. He was managed with antibiotics, analgesics, and low-dose prednisolone (1 mg/kg/day) for seven days. He was discharged in stable condition and seen for follow-up two weeks later with complete resolution of the scrotal swelling and purpura. Subsequent follow-up by the nephrology team with urine protein/creatinine ratio, albumin/creatinine ratio, renal function, and liver function tests were all normal, thus excluding long-term renal sequelae (Figures 1, 2).

The decision to do an urgent scrotal exploration to exclude testicular torsion was taken. The intraoperative finding was that of a swollen oedematous epididymis and testis. There was no evidence of testicular torsion (Figures 1, 2).

## Discussion

Henoch-Schönlein Purpura (HSP), now known as Immunoglobulin A vasculitis (IgAV), is the most common form of systemic vasculitis in children, primarily affecting those between the ages of 3 and 15 years. The incidence of HSP varies widely, with reported rates ranging from 3 to 27 cases per 100,000 children annually [1,5]. It is more prevalent in boys than girls and is seen more frequently in the autumn and winter months. Although HSP can occur in adults, it is relatively rare and tends to present with more severe kidney involvement in this population [1,7,8].

IgAV is distinguished by a classic tetrad of clinical manifestations characterised by purpuric rash, arthralgia, abdominal pain, and kidney involvement. The rash is the hallmark of the disease, characterised by small, raised areas of bleeding under the skin, typically appearing on the lower extremities and buttocks. These purpuric lesions are palpable and usually do not blanch on pressure. They may be itchy but rarely painful. 

Joint involvement is common, especially in the knees and ankles, leading to pain, swelling, and limited movement. The arthritis is typically transient or migratory, involving one or more joints. Gastrointestinal symptoms may include intermittent abdominal pain, which can be severe and mimic other acute abdominal conditions. The pain is typically colicky and may be associated with gastrointestinal bleeding, while kidney disease can range from mild to severe and is often indicated by haematuria (blood in the urine) and proteinuria (protein in the urine). Though rare in children, severe kidney involvement is more commonly seen in adults with this condition.

Involvement of other parts of the urogenital system is less common, but no less significant. The involvement of the scrotum, ureter, bladder, prostate, testicles, and penis has all been reported. Scrotal or testicular involvement is rare [7-9]. They may present with acute scrotum, epididymitis, and orchitis. Scrotal involvement associated with HSP typically manifests as scrotal pain, swelling, and erythema. These symptoms may manifest at the same time as the HSP symptoms or may arise before the symptoms or after the resolution of HSP symptoms [3]. Thus, diagnosis is often missed and requires a high index of suspicion. Recent evidence suggests that widespread purpura, local oedema, penile involvement, and haematuria are more common in patients with scrotal involvement than those without, but there’s no evidence to suggest that this may be associated with a worse clinical outcome [10]. The index case in this report presented as a case of acute scrotum with non-specific abdominal pain and a classical purpuric rash after resolution of most other symptoms. Diagnosis of HSP is often clinical if patients present with classical features, which include palpable purpura on the lower limbs and buttocks, arthritis of the hips, knees, and ankles, and abdominal pain. Occasionally, diagnosis may be less clear, requiring skin biopsies to examine for IgA deposition. A large amount of IgA deposits in the organ being tested favours the diagnosis of HSP [5,11]. Scrotal duplex Doppler sonography and radionuclide scanning may be helpful in excluding testicular torsion. However, this should not delay scrotal exploration in equivocal cases. This index patient presented out of hours, and scrotal Doppler sonography was not immediately available. A similar presentation related to this case has previously been reported by previous authors [12]. Sonographic findings in keeping with HSP include hydrocele, swelling in the epididymis and scrotal skin, and normal or increased blood flow to the testes. Treatment of HSP is largely symptomatic and primarily supportive and includes hydration, bed rest, and analgesia. Severe cases may require steroids and immunosuppressive therapy. Similarly, treatment of scrotal involvement varies from conservative to immunosuppressive therapy, and steroids have been shown to be very useful in patients with scrotal involvement [10-13].

## Conclusions

Testicular manifestation of HSP is rare and can mimic an acute scrotum. A high index of suspicion is required for prompt diagnosis. HSP-related epididymo-orchitis should be suspected in a male child presenting with acute scrotal pain and non-blanching purpura of the lower extremities. Treatment is mainly symptomatic and supportive. However, scrotal exploration may be required in equivocal cases.
